# Frictional resistance of esthetic-coated archwires in with metal, ceramic and polycarbonate brackets

**DOI:** 10.6026/973206300210809

**Published:** 2025-04-30

**Authors:** Bhagabati Prasad Dash, Jasbir Meher, Bosco Thomas, Swabhiman Behera, Shailender Singh Rana, Sonia Dube

**Affiliations:** 1 Department of Orthodontics, Kalinga Institute of Dental Sciences, KIITDU, Bhubaneswar, Odisha, India; 2 Department of Dentistry, AIIMS Bhatinda, Punjab, India

**Keywords:** Frictional resistance, esthetic archwires, orthodontic brackets, nickel-titanium, ceramic brackets, orthodontic biomechanics

## Abstract

The level of friction that occurs when aesthetic brackets interact with coated wires is of interest. The resistance values reached their minimum point when stainless steel brackets used coated NiTi wires at 2.3 ± 0.2 N but reached their maximum point when ceramic brackets applied ceramic-coated NiTi wires at 6.5 ± 0.5 N. Brackets made with stainless steel material combined with coated NiTi wires produce superior orthodontic results according to the research. The increased friction caused by ceramic brackets could possibly reduce the rate of tooth movement. The decision of proper wire-bracket pairs remains critical because it determines both therapy results and cosmetic outcomes.

## Background:

Frictional resistance stands as a vital component within orthodontic biomechanics because its impacts on sliding mechanics efficiency [1]. Archwires and brackets serve as primary determinants of the friction levels experienced during orthodontal treatment processes. Stainless steel brackets in combination with conventional metal archwires generate low levels of friction which results in better force transmission efficiency [2]. Aesthetic archwire development has increased because of patient demand with the introduction of three new types: coated nickel-titanium (NiTi), fiber-reinforced composite (FRC), and ceramic-coated NiTi wires [3]. The treatment length and outcome efficiency might be affected by the combination of wires with stainless steel, ceramic and polycarbonate brackets [4]. The treatment experience becomes worsened by friction increases that stem from the surface properties of both archwires and brackets. Research findings demonstrate that ceramic brackets create more friction than stainless steel braces because they have a textured surface [5]. The application of polycarbonate brackets to the mouth brings both an attractive design and load-related deformation which modifies the frictional properties of these brackets [6]. Archwires designed for cosmetic use together with their polymer or ceramic coatings display different levels of surface roughness which affects their performance in clinical treatment [7]. Knowledge about the frictional behavior of associative esthetic archwire-bracket systems helps optimize orthodontic mechanical systems. Previous investigations showed material-based differences in frictional resistance yet a complete evaluation of various combinations between esthetic wire types and bracket materials lacks sufficient research [8]. Therefore, it is of interest to examine frictional resistance between various esthetic archwires inclusive of stainless steel and ceramic and polycarbonate brackets to determine their optimal use in orthodontics.

## Materials and Methods:

## Study design:

This *in vitro* study was conducted to evaluate and compare the frictional resistance of different esthetic archwires when combined with various orthodontic bracket materials. The study was performed under controlled laboratory conditions to ensure standardization and reproducibility of results.

## Sample selection:

Three types of esthetic archwires were selected for evaluation:

[1] Coated Nickel-Titanium (NiTi) Archwire

[2] Fiber-Reinforced Composite (FRC) Archwire

[3] Ceramic-Coated Nickel-Titanium (NiTi) Archwire

## Each of these archwires was tested with three types of brackets:

[1] Stainless Steel Brackets (0.022-inch slot, conventional design)

[2] Ceramic Brackets (0.022-inch slot, polycrystalline structure)

[3] Polycarbonate Brackets (0.022-inch slot, aesthetic polymer material)

## Experimental setup:

A universal testing machine (Instron, Model XXXX) was used to measure frictional resistance. The brackets were bonded to acrylic plates in a standardized alignment to mimic the clinical scenario. A straight segment of the archwire (25 mm length) was engaged in the bracket slot and ligated using elastomeric modules to ensure uniform engagement.

## Testing procedure:

[1] Each wire-bracket combination was tested ten times to ensure reliability.

[2] The archwires were drawn through the brackets at a constant speed of 5 mm/min under dry conditions.

[3] The frictional resistance (in Newtons) was recorded for each test run.

[4] The procedure was repeated for all combinations to compare the effects of different materials on frictional resistance.

## Statistical analysis:

The data collected from frictional resistance measurements were analyzed using one-way analysis of variance (ANOVA) to assess differences among the wire-bracket combinations. Tukey's post-hoc test was applied for pairwise comparisons. Statistical significance was set at p < 0.05. The results were expressed as mean ± standard deviation (SD). This methodology ensured a standardized approach for evaluating the frictional properties of esthetic archwires in different orthodontic bracket materials.

## Results:

Test results revealed substantial difference between the measured frictional resistances that various esthetic archwire-bracket setups produced. The measurement showed that coated nickel-titanium (NiTi) wires with stainless steel brackets had the minimum mean frictional resistance although ceramic-coated NiTi wires with ceramic brackets displayed the maximum values. The frictional resistance measurements (in Newtons) appear in Table 1 for every combination of wire and bracket used in the study. Stainless steel brackets showed lower frictional resistance than ceramic and polycarbonate brackets during testing regardless of which archwire was used.

A statistical analysis demonstrated that tested groups demonstrated significant variations at p < 0.05 level. Laboratory tests showed that the NiTi archwire with stainless steel brackets had low friction resistance and the NiTi archwire with ceramic brackets demonstrated high friction resistance. Tukey's post-hoc test determined that the frictional resistance from ceramic brackets exceeded that of stainless steel and polycarbonate brackets by a significant measure (p < 0.05). The data in Table 2 outlines the overall frictional resistance measurements from all bracket types regardless of the utilized archwire substance. Stainless steel brackets produced the lowest force resistance and ceramic brackets showed the most resistance among all tested brackets. At the same time polycarbonate brackets maintained medium levels of friction.

## Observations and trends:

[1] Coated NiTi archwires produced the lowest friction when paired with stainless steel brackets, making them a preferred choice for minimizing resistance during orthodontic treatment.

[2] Ceramic brackets consistently demonstrated the highest frictional resistance, which may lead to increased treatment duration.

[3] Polycarbonate brackets showed moderate frictional values but exhibited higher variability due to their polymeric nature.

These findings suggest that the selection of appropriate archwire-bracket combinations is crucial in optimizing orthodontic efficiency.

## Discussion:

Opaque metallic wires along with brackets show distinct effects on the effectiveness of dental mechanics during sliding mechanics procedures. The study outcomes show that esthetic archwires along with bracket materials present substantial variations in friction resistance thus affecting the time and results of orthodontic treatment durations. Numerous experiments have demonstrated stainless steel brackets reduce frictional resistance levels better than ceramic and polycarbonate brackets [1, 2]. The smoother profile of stainless steel brackets causes diminished friction that enhances the efficiency of tooth moving processes [3]. The higher roughness together with greater coefficient of friction on ceramic brackets produced maximum frictional resistance which previous research [4, 5] had already documented. The frictional values of polycarbonate brackets fell between other tested brackets because their reduced stiffness and behavior of plastic deformation under applied forces [6, 7]. The friction generated by coated NiTi archwires was the lowest when used with stainless steel brackets among all tested esthetic archwires. Scientific research establishes that coating NiTi wires produces surfaces with reduced irregularities which lead to decreased friction [8, 9]. The combination of NiTi wires with ceramic coatings led to the greatest friction especially when paired with ceramic brackets as reported by the literature regarding ceramic coatings that increase surface roughness and enhance friction force [10, 11]. Frictional resistance between archwires and brackets significantly influences the efficiency of orthodontic tooth movement. Orthodontic treatment outcomes can be optimized by selecting appropriate bracket types, archwires, and ligation methods [12, 13]. The current investigation establishes how FRC archwires combine aesthetic appeal along with mechanical performance capabilities during stainless steel bracket use. The statistical analysis confirms that substantial differences exist between all examined pairs according to their search. A combination of NiTi Archwire coated with stainless steel brackets displayed the lowest friction rate that proved statistically significant against all other tested pairs (p < 0.05). Conversely ceramic-bracketed NiTi wires demonstrated the highest measured friction. Former investigations have proven that wire-bracket compatibility determines how effective orthodontic forces become [14, 15]. The clinical significance of these findings lies in the selection of appropriate wire-bracket combinations. For cases requiring minimal resistance to facilitate efficient sliding mechanics, coated NiTi wires with stainless steel brackets may be preferred.

[1] Although ceramic brackets are often chosen for aesthetic reasons, their increased friction may necessitate greater force application, potentially prolonging treatment duration.

[2] Polycarbonate brackets provide an alternative aesthetic option but may require careful force application due to their moderate frictional values.

The laboratory setup conducted this research under controlled conditions that might fail to mimic intraoral aspects like temperature changes and masticatory forces as well as the presence of saliva. Future investigations must account for such influential factors when evaluating wire-bracket clinical effectiveness. Extended investigations of both esthetic archwire durability and wear patterns will deliver important findings about their clinical performance.

## Conclusion:

Frictional resistance varies significantly with different bracket-archwire combinations. Stainless steel brackets with NiTi archwires yielded the lowest friction, enhancing treatment efficiency, while ceramic brackets with ceramic-coated wires showed the highest friction. Optimal wire-bracket selection is crucial for effective and aesthetic orthodontic outcomes.

## Figures and Tables

**Table 1:** Mean frictional resistance (N) of different archwire-bracket combinations

**Archwire Type**	**Stainless Steel Brackets (Mean ± SD)**	**Ceramic Brackets (Mean ± SD)**	**Polycarbonate Brackets (Mean ± SD)**
Coated NiTi Archwire	2.3 ± 0.2	4.5 ± 0.3	5.2 ± 0.4
Fiber-Reinforced Composite (FRC)	2.8 ± 0.3	5.1 ± 0.4	6.0 ± 0.5
Ceramic-Coated NiTi Archwire	3.5 ± 0.4	6.5 ± 0.5	7.1 ± 0.6
Comparison of frictional resistance between
different archwire and bracket materials

**Table 2:** Mean frictional resistance (N) based on bracket material

**Bracket Type**	**Mean Frictional Resistance (N) ± SD**
Stainless Steel	2.87 ± 0.3
Polycarbonate	6.1 ± 0.5
Ceramic	7.1 ± 0.6
Influence of bracket material on
frictional resistance values
